# Predicting tachycardia as a surrogate for instability in the intensive care unit

**DOI:** 10.1007/s10877-019-00277-0

**Published:** 2019-02-14

**Authors:** Joo Heung Yoon, Lidan Mu, Lujie Chen, Artur Dubrawski, Marilyn Hravnak, Michael R. Pinsky, Gilles Clermont

**Affiliations:** 1 Division of Pulmonary, Allergy, and Critical Care Medicine, Department of Medicine, University of Pittsburgh, Pittsburgh, PA USA; 2 Department of Critical Care Medicine, University of Pittsburgh, Pittsburgh, PA USA; 3 Auton Lab, Department of Computer Science, Carnegie Mellon University, Pittsburgh, PA USA; 4 School of Nursing, University of Pittsburgh, Pittsburgh, PA USA; 5 2557 Terrace Street, 6th Floor, Pittsburgh, PA 15206 USA

**Keywords:** Prediction, Tachycardia, Intensive care unit, Critical Care, Machine learning

## Abstract

Tachycardia is a strong though non-specific marker of cardiovascular stress that proceeds hemodynamic instability. We designed a predictive model of tachycardia using multi-granular intensive care unit (ICU) data by creating a risk score and dynamic trajectory. A subset of clinical and numerical signals were extracted from the Multiparameter Intelligent Monitoring in Intensive Care II database. A tachycardia episode was defined as heart rate ≥ 130/min lasting for ≥ 5 min, with ≥ 10% density. Regularized logistic regression (LR) and random forest (RF) classifiers were trained to create a risk score for upcoming tachycardia. Three different risk score models were compared for tachycardia and control (non-tachycardia) groups. Risk trajectory was generated from time windows moving away at 1 min increments from the tachycardia episode. Trajectories were computed over 3 hours leading up to the episode for three different models. From 2809 subjects, 787 tachycardia episodes and 707 control periods were identified. Patients with tachycardia had increased vasopressor support, longer ICU stay, and increased ICU mortality than controls. In model evaluation, RF was slightly superior to LR, which accuracy ranged from 0.847 to 0.782, with area under the curve from 0.921 to 0.842. Risk trajectory analysis showed average risks for tachycardia group evolved to 0.78 prior to the tachycardia episodes, while control group risks remained < 0.3. Among the three models, the internal control model demonstrated evolving trajectory approximately 75 min before tachycardia episode. Clinically relevant tachycardia episodes can be predicted from vital sign time series using machine learning algorithms.

## Introduction

In modern ICU environments, tachycardia is one of the most common and earliest vital sign responses in critically-ill patients to impending cardiorespiratory instability (CRI), [1] as it parallels increased sympathetic nervous system activity. Tachycardia also reflects the normal physiologic response of the body’s effort to maintain cardiac output and thereby meeting metabolic demand of vital organs when delivery is compromised as a consequence of a variety of pathological processes such as decreased stroke volume (e.g. hypovolemia, cardiac pump dysfunction, pulmonary embolism), decreased oxygen carrying capacity (e.g. hypoxia, anemia), or decreased arterial tone (e.g. vasoplegia, sepsis). Tachycardia is an independent risk factor linked to worse outcomes for several different chronic conditions including heart failure [2, 3] and coronary artery disease [4–6]. Tachycardia may independently contribute to worse outcome in acutely decompensated states, not only of cardiac origin (e.g. post-myocardial infarction [7], cardiogenic shock [8–10]), but also in shock of other etiologies with obstructive (e.g. pulmonary embolus [11, 12]) or distributive physiology (e.g. sepsis [13–15]). In addition, tachycardia correlates with worse post-operative outcome, as reflected in the Modified Early Warning System (MEWS) score, where tachycardia correlates with mortality [16]. Thus, early detection and/or prediction of impending tachycardia, although in itself a non-specific indicator of stressed physiology, could lead to earlier detection, and potentially earlier interventions to rescue patients with impending CRI.

A major challenge in predicting CRI from complex physiologic time series data is the ability to transform those data into a reliable risk model. Recently, data-driven classification methods with parsimonious use of multi-granular features show promise in understanding embedded patterns from complex vital sign trends preceding overt CRI [17]. Our group has previously shown the utility of a composite early warning signature vital sign index in the early prediction of CRI in step-down patients, wherein upcoming CRI events were predicted with an accuracy of 80% at 9.7 min prior to overt instability [18]. We have also shown variations among risk trajectories leading up to CRI [19]. Accordingly, we hypothesized that tachycardia, as a surrogate early instability marker, could be reliably predicted in ICU patients using featurized vital sign trends, and that there exists a finite variety of risk trajectories among patients leading up to tachycardia.

## Methods

### Source data

The study used the Multiparameter Intelligent Monitoring in Intensive Care II (MIMIC-2, version 26) database; a free, publicly-available comprehensive multi-granular database constructed from all intensive care visits at a tertiary care hospital in Boston, MA from 2001 to 2007 [20]. MIMIC-2 includes data from 25,328 ICU visits, segregated across three domains. The clinical database includes demographic data, lab and medication data, and image as well as text data such as physician’s records and nursing notes. It is thus an abridged version of the electronic-health record. The numerical database includes a collection of frequently sampled (1/60 Hz or 1 Hz) vital signs data, including heart rate, blood pressure, respiratory rate, temperature, and oxygen saturation from pulse oximetry. The waveform domain, not used in the current study, includes waveforms (125–250 Hz) from electrocardiogram (EKG) monitors, blood pressure monitored from the arterial line, or changes in breathing and oxygen saturation directly recorded by photoplethysmography in a subset of subjects. To utilize the multi-granular character of the dataset, only subjects with overlapping data across all three domains (clinical, numerical, and waveform) were considered. We did not specify the type of ICU for our analysis, but restricted the subject age to be greater than 18 years old.

### Operational definition of tachycardia episodes

Traditionally, physiologic tachycardia has been defined as a heart rate (HR) greater than 100 beats/min [21]. Tachycardia correlates with worse prognosis, in both acutely decompensated [16] and chronically compensated states [22]. Using MEWS, one study associated HR > 129/min in post-operative patients with significantly increased mortality [16]. Similarly, the Royal College of Physician suggested the National Early Warning Score (NEWS) to assess acute illness in pre-hospital and hospital settings. In the NEWS schema, a HR of 130/min indicated greatest risk and mandated a ‘step change’ in acute care management protocol [23]. We further validated the use of this threshold of 130/min in our study population as described below.

**Fig. 1:**
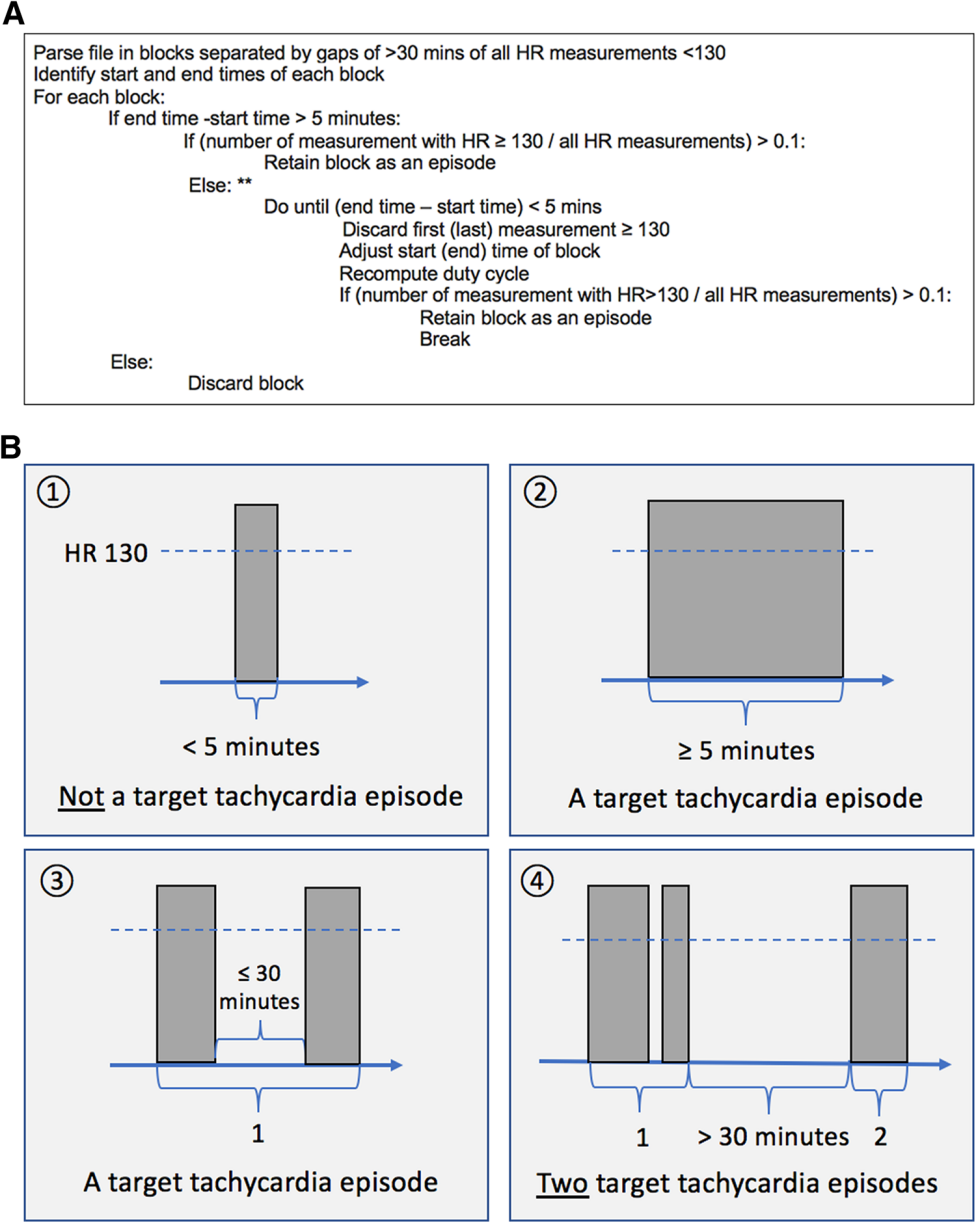

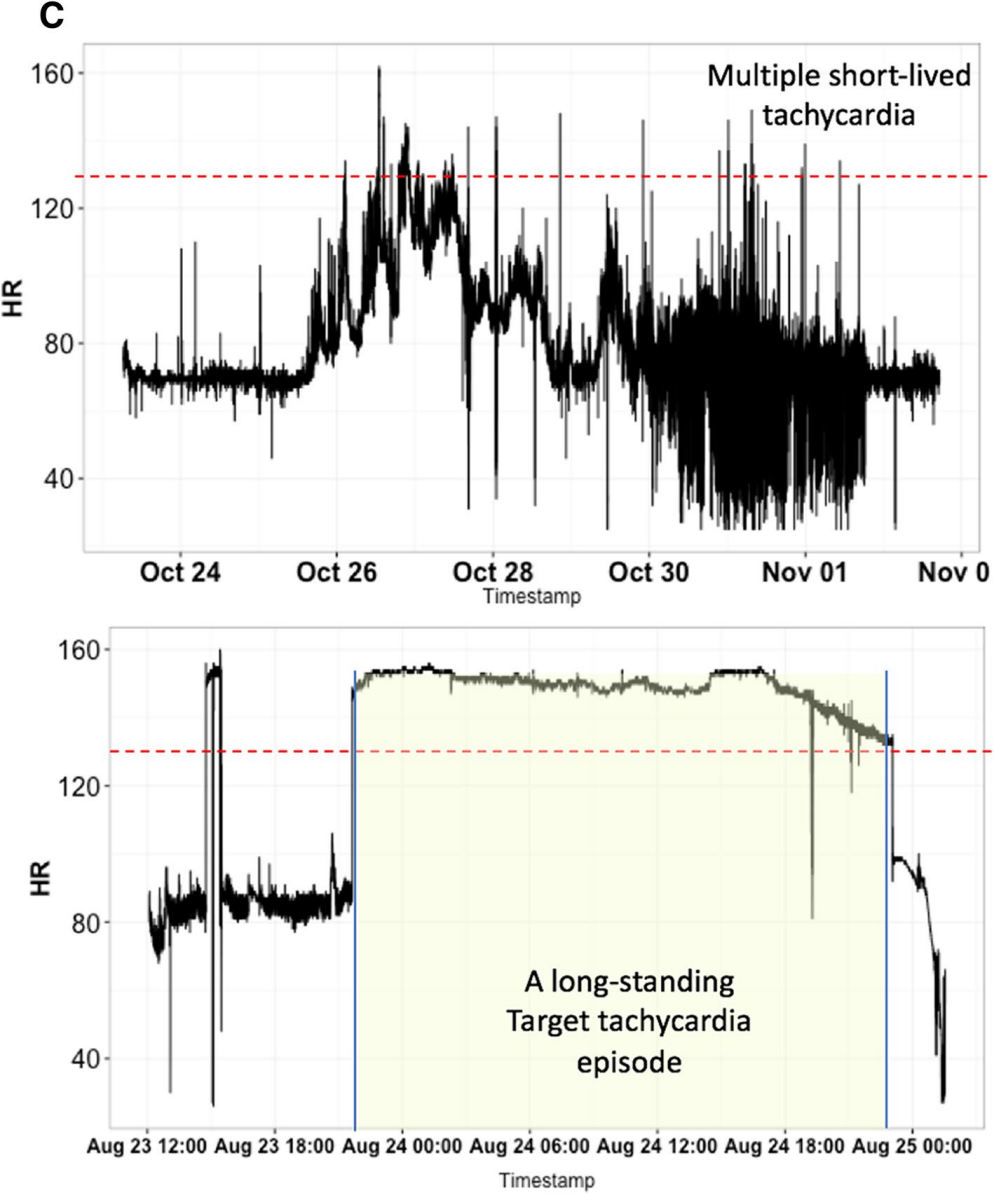
Operational definition of target tachycardia episode. **a**. A pseudocode for selecting target tachycardia episode by operational definition. **b** Schematic illustration of tachycardia episodes by rate, length, and interval. The time between events less than 30 min were combined to form an episode. **c** An illustration for the concept of density (‘duty cycle’) with two examples of heart rate time series. Red dotted lines indicate the threshold for tachycardia for each episode, with shaded area on the bottom graph shows the time period satisfying the operational definition of a tachycardia episode. Note while upper graph showed much larger number of episodes, lower graph revealed a single, but much dense episode of continued tachycardia. The subject in the bottom panel eventually expired in the ICU

We then proceeded to define tachycardia episodes as depicted in Fig. 1a. Using the numerical HR data (1/60 Hz or 1 Hz), periods of > 30 min without instances of HR > 130/min separated potential episodes (Fig. 1b). Potential episodes were further processed to retain only periods of time lasting at least 5 min and where at least 10% (duty cycle of ≥ 10%) of measurements were ≥ 130/min (Fig. 1c).

### Clinical relevance of target tachycardia episodes

To support the clinical relevance of this operational definition of tachycardia episode as a legitimate target for prediction, we evaluated use of norepinephrine, incidence of hypotension, transfusion of red blood cells, length of ICU stay, and all-cause mortality at thresholds of tachycardia of 110/min, 120/min, and 130/min, and compared outcome to a control group never experiencing tachycardia (HR < 110/min) during their ICU stay.

### Heterogeneity of tachycardia episodes

There are distinct mechanisms of tachycardia: sinus tachycardia, atrial fibrillation, ventricular fibrillation, and several others. To support our decision to group all episodes of tachycardia as a single target for prediction, we wished to demonstrate that different mechanisms were associated with uniformly poorer outcomes compared to the non-tachycardic subjects. We identified subjects with atrial fibrillation or ventricular tachycardia if they received metoprolol, amiodarone, diltiazem, xylocaine and digoxin within 3 h of onset of tachycardia as a separate group. We compared the aforementioned outcomes from this group to subjects with tachycardia not receiving these drugs in the 3 h window, and to non-tachycardic subjects.

### Tachycardia case and control groups

**Fig. 2:**
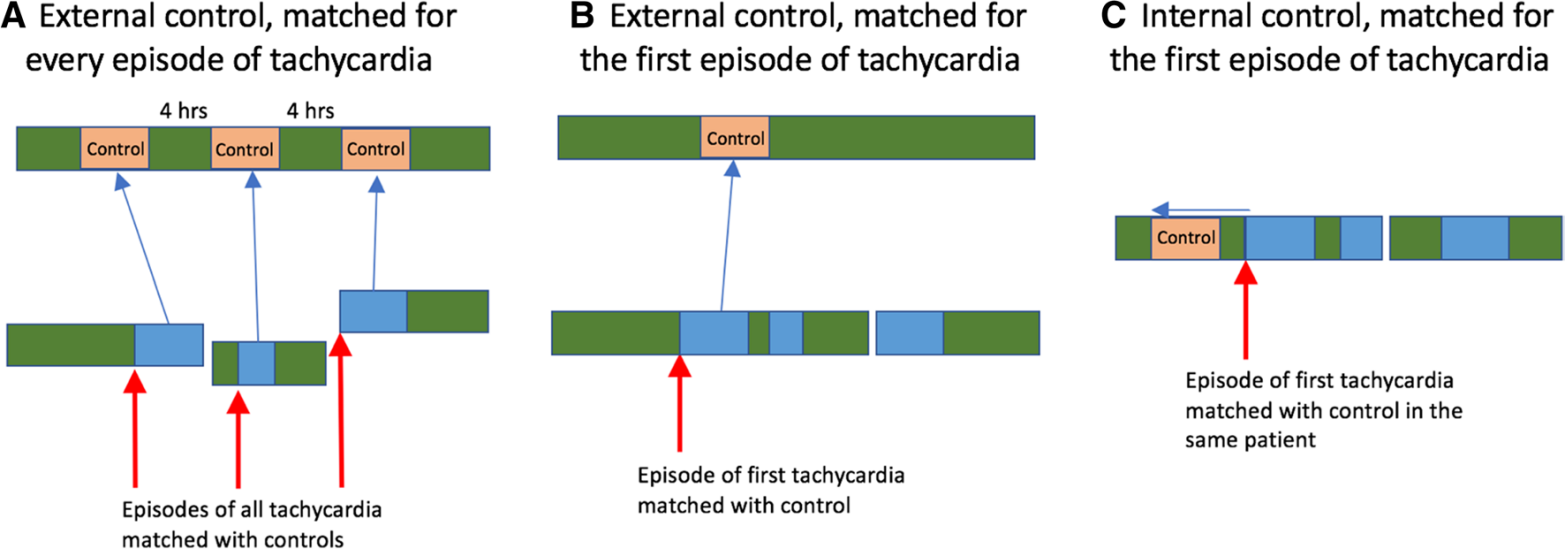
The three models for tachycardia episodes with corresponding control groups

Subjects who fulfilled study entry criteria but never exhibited tachycardia during their ICU stay comprised the control subjects. For each control subject, we randomly selected a 30-min time window during their ICU stay to construct a set of control windows. For each subject with at least one episode of tachycardia, we selected a 30-min time window immediately prior to onset of every tachycardia episode to construct a set of case windows with 1:1 patient matching. We developed three different models using the following choices of cases and control windows (Fig. 2). The first modeling group (Group 1) used all case periods and a random subset of control periods so as to produce an approximate 1:1 match between cases and controls. In group 2, we only considered the first tachycardia episode from each case subject, and the same control windows as Group 1 are used. In Group 3, case windows were identical to Group 2, but control windows were selected from the same subjects as cases, from a 3 h period well in advance of developing tachycardia (internal controls). Models developed in Groups 1 and 2 tested whether the first episode of tachycardia was easier to predict than any episode of tachycardia, while Group 3 tested whether subjects who develop tachycardia display discernible trends in risk scores, irrespective of absolute risk.

### Model learning and risk trajectories

**Fig. 3:**
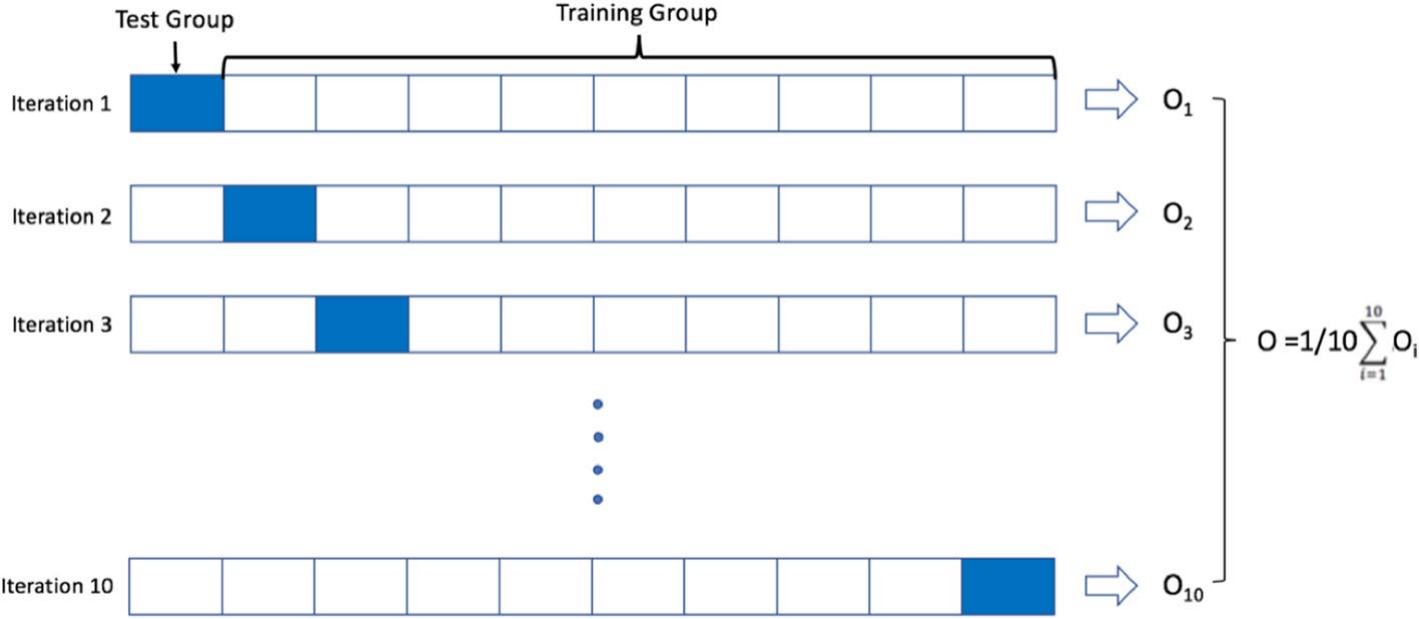
Ten-fold cross validation method for training and test models

We computed a set of features to characterize 30-min windows of vital sign times series data. For cases, the data window was immediately prior to the tachycardia episodes. For controls, features were computed from a random 30-min window of data. We then produced a tachycardia risk score using regularized lasso logistic regression (LR) and random forest (RF) [24–28]. The risk score was set to range from 0 (lowest risk) to 1 (highest risk) and can be interpreted as the probability of tachycardia in the next minute, given the last 30 min of data. Both algorithms were trained using a 10-fold cross validation method to mitigate overfitting [29]. In detail, for each prediction model, groups were divided into 10 equal size subgroups. Nine out of ten subgroups were used to train the model. The remaining subgroup was used for testing. The procedure is repeated ten times using a different testing subgroup each time. Results were pooled across the test subgroups to express the performance of the model (Fig. 3). Receiver operating characteristic (ROC) curves was used as performance metric.

Then, risk trajectories were developed for the three modeling groups. To generate a subject’s instantaneous risk score, we computed the model’s predicted risk the preceding 30 min of data. Risk scores were updated every minute to create a risk score trajectory covering the entire duration of the data stream in each subject, except for the first 30 min. We also wished to understand the ability of some predictors to forecast tachycardia with longer lead times. We thus constructed “lagged” models, where the lag is the number of minutes between the time window and the onset of tachycardia (For example, a lag-30 model used data from a window between − 60 min and − 30 min to the episode). For Group 3, we used a concept called ‘lift score’. A subgroup of case subjects with at least 3 h of data without a tachycardia episode prior to the first episode was selected. An average, patient-specific, risk score was computed over this entire 3-h data segment. Then the minute-by-minute risk score from 3-h prior to tachycardia episode for each patient was divided over the average baseline risk score for each patient to calculate the lift score.

Data were pre-processed using Python version 3.4 (Python Software Foundation, Wilmington, DE) and of the data was done using Matlab version r2016b (MathWorks, Natick, MA) was used. Machine learning work was performed using the scikit-learn Python library and customized software [30].

## Results

### Clinical relevance of tachycardia episodes

**Fig. 4:**
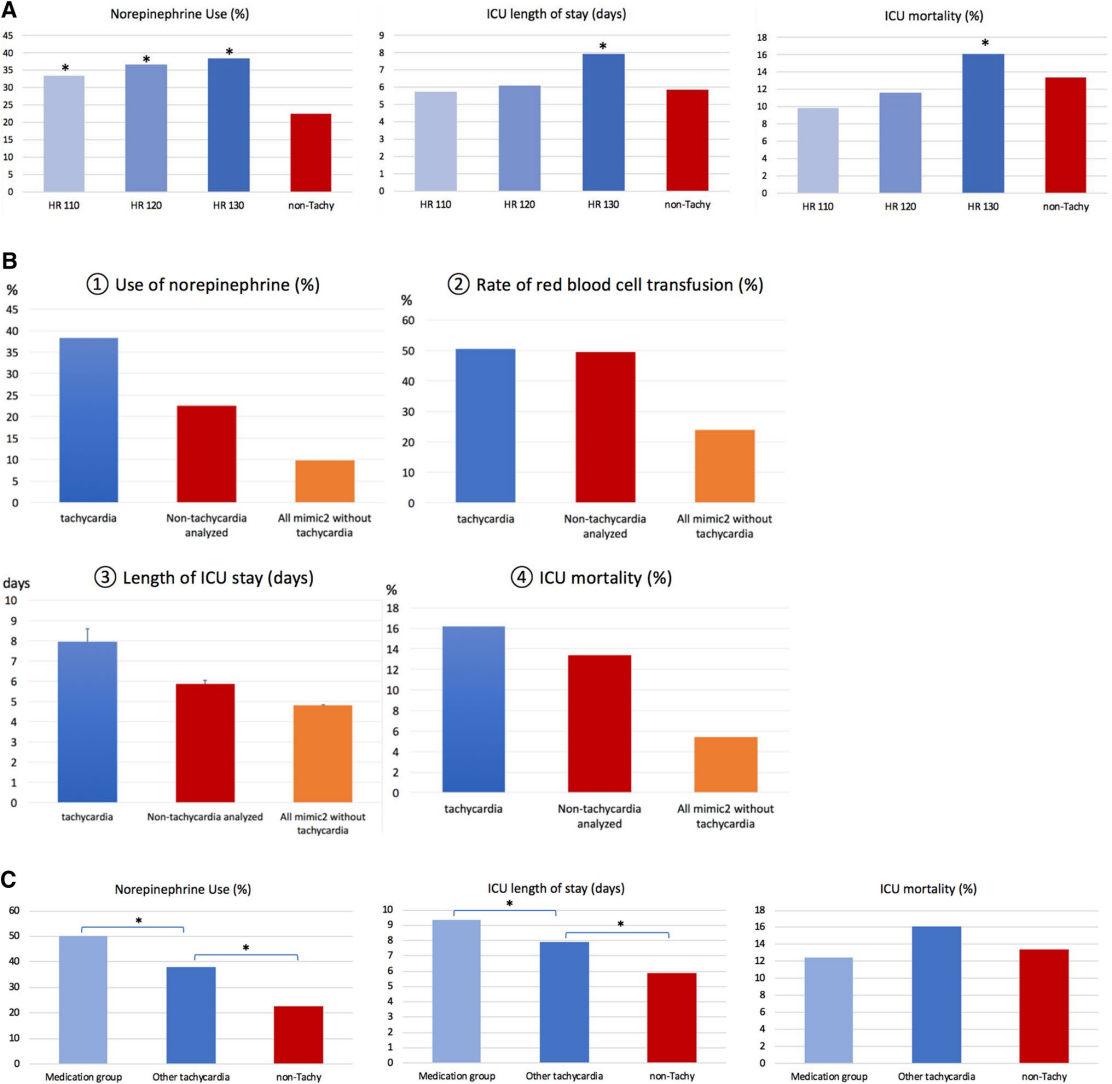
Clinical relevance of target rate for tachycardia episode. **a** Selection of rate thresholds for target tachycardia episode. With using heart rate (HR) 110/min, 120/min, and 130/min cut-off, different adverse clinical outcome variables including the use of norepinephrine (%), ICU length of stay (days), and ICU mortality (%). Non-tachycardia comprises control group which had no tachycardia episode during ICU stay (n = 2376). **b**. Clinical adverse outcomes for tachycardia subjects who met operational definition ‘Tachycardia’ indicates subjects met operational definition of tachycardia during the ICU stay (n = 235). ‘Non-tachycardia analyzed’ subjects are control group without tachycardia episode (rate threshold of HR < 130) during the ICU stay (n = 2572). ‘All mimic2 without tachycardia’ stands for the rest of MIMIC2 patient (n = 39397). When appropriate, mean and the standard error (SEM) was produced with error bars. **c** Comparison of clinically important abrupt onset of non-sinus tachycardia episodes with other overall tachycardia episodes as well as non-tachycardia MIMIC 2 dataset

Tachycardia thresholds of HR ≥ 110/min (n = 428), 120/min (n = 328) and 130/min (n = 235) were associated with increasing incidence of norepinephrine use of 33.4% (143/428), 36.8 (121/328) and 38.3% (90/235) respectively. Although this trend was not significant across tachycardia groups, all were higher than norepinephrine use in the non-tachycardia group (n = 2376) defines as HR < 110/min (22.1%). In particular HR > 130/min was significantly different than HR < 110/min (p = 0.009) in their use of norepinephrine. Subjects with HR ≥ 110/min had an ICU length of stay of 5.73 days whereas those with HR ≥ 120/min had an average stay of 6.12 days, showing no statistical significance compared to non-tachycardic control group (5.85 days). However, subjects with HR ≥ 130/min group (7.94 days) showed statistically longer length of stay compared to the non-tachycardia group. There was a trend between degree of tachycardia and ICU mortality, which did not reach statistical significance (Fig. 4a).

The incidence of red blood cell transfusion was not different between the tachycardia case and control groups (Fig. 4b) (50.6% vs. 49.4%, p = 0.724). In addition, relationships with other direct CRI target variables were investigated. Among the tachycardia group, we found hypotension followed the first tachycardia episodes in 49% subjects (115 out of 235) within next 24 h. Whereas hypotension without tachycardia episodes developed in only 7% of subjects (174 out of 2572). We identified 22 instances of intravenous medication administration within 3 h of the onset of a tachycardia episode (22/787, 2.8%) in 8 subjects (8/235, 3.4%). As summarized in Fig. 4c, when compared with tachycardia subjects as well as non-tachycardia subjects, those received intravenous antiarrhythmic medications within 3 h after the onset of tachycardia episodes (‘medication group’) showed increased use of norepinephrine (4 out of 8 subjects, 50%) and longer ICU stay (9.32 vs. 7.94 days). All-cause ICU mortality, however, showed no statistically significant difference (1 out of 8, 12.5%) compared with tachycardia subjects in general (16.1%). Both subjects in the medication group and subjects with tachycardia, but who did not belong to the medication group, has highly significantly different rate of norepinephrine use (p < 0.001) and ICU length of stay (p = 0.003).

### Selection of tachycardia episodes and control group

A total of 2809 subjects were found to have data identifier across all three (clinical, numerical, and waveform) databases, from which 787 episodes of tachycardia episodes were identified in 240 subjects. A randomly selected set of 240 non-tachycardic subjects generated 707 control windows devoid of artifacts. The median length of target tachycardia episode was found to be 1407 s (23.45 min) with standard deviation of 16968 s (4.71 h). The average density (duty cycle) of target tachycardia episode was 35%, and its standard deviation was 27% (minimum 10.01%, maximum 100%). Various clinical characteristics for tachycardia and control groups are summarized on Table 1. Tachycardia group tends to be older in age at admission to ICU (72 vs. 65.7, p = 0.02) and clinically associated with higher mortality with Elixhauser criteria (11.34 vs. 9.43, p < 0.01). The ICU length of stay was also longer in tachycardia group (7.9 vs. 5.6 days, p = 0.02).

**Table 1:** Demographic characteristics for tachycardia and control group subjects

Variables	Tachycardia group	Control group	p-values
Age (years)	72 ± 31.8	65.7 ± 27.3	0.02
Gender (male)	52.5%	59.5%	0.58
Body Mass Index	28.6 ± 8.6	29.1 ± 8.8	0.57
First SOFA score	6.4 ± 4.6	5.8 ± 4.3	0.16
Maximum SOFA score	7.7 ± 4.8	6.9 ± 4.2	0.38
Elixhauser score	11.34 ± 7.66	9.43 ± 7.89	< 0.01
First ICU types			
Cardiac	95 (39.6%)	106 (44.2%)	0.31
Cardiothoracic	105 (43.8%)	98 (40.8%)	0.58
Medical	16 (6.7%)	18 (7.5%)	0.72
Others	23 (9.6%)	18 (7.5%)	0.51
ICU length of stay (days)	7.9 ± 9.9	5.9 ± 9.26	0.02
ICU mortality (%)	15.8	12.9	0.43

### Featurization, model development, and assessment

A total of 42 vital sign features were generated and provided as candidate predictors to the ML algorithms (Table 2). Main features included approximate entropy, autocorrelation, and fast Fourier transform sum of squared amplitudes, along with a variety of statistical features such as mean and standard deviation. Approximate entropy provides the unpredictability of fluctuations in a given time series. However, this would be dependent upon the patterns of length and inherent similarity. Autocorrelation measures the correlation between a signal and time-lagged instances of the signals, therefore quantifying memory in the system. Rapid decreases in autocorrelation with increasing time lags indicates short memory in the system. In using the discrete Fourier transform, we used cubic-spline interpolation of heart rate and respiratory rate data for missing data as long as at least 20% of the data were available. The final feature used was the sum of spectral amplitudes, where frequencies ranged from 0 to 1/120 Hz.

**Table 2:** List of predictors for tachycardia episode

Feature abbreviation	Feature name	Remarks
mean_abpdias mean_abpmean mean_abpsys mean_hr mean_rr mean_spo2	Mean values for vital signs	
sd_hr sd_rr sd_spo2	Standard deviations for vital signs	
reg_ abpdias reg _abpmean reg _abpsys reg _hr reg _rr reg _spo2	Coefficient of first-order regression	Degree of association between the two predictor variables
fft_hr fft_rr fft_spo2	Fast Fourier transformation	Converts a signal from its original domain to a representation in the frequency domain
acs_hr acs_rr acs_spo2	Autocorrelation	Measures the degree of similarity between a given time series and its lagged version over continuous time periods
aes_hr aes_rr aes_spo2 ses_hr ses_rr ses_spo2	Approximate entropy Sample entropy	Reflects the likelihood that similar patterns of observation will not be followed by additional similar observations
density_hr density _rr density _spo2	Density of the records	The amount of data points available during the given time interval
last_5min_mean_hr last_5min_mean_rr last_5min_mean_spo2	Mean value in the last 5 min	Considering the rapidly deteriorating conditions during last 5 or 10 min prior to the instability, short-term means and coefficients were separately assessed and applied
last_5min_reg_hr last_5min_reg_rr last_5min_reg_spo2	Coefficients of the first-order regression in the last 5 min	
last_10min_mean_hr last_10min_mean_rr last_10min_mean_spo2	Mean value in the last 10 min	
last_10min_reg_hr last_10min_reg_rr last_10min_reg_spo2	Coefficients of the first-order regression in the last 10 min	

*Abpdias* diastolic arterial blood pressure; *abpmean* mean arterial pressure; *abpsys* systolic arterial blood pressure; *acs* autocorrelation; *aes* approximate entropy; *fft* fast Fourier transformation; *hr* heart rate; *min* minutes; *reg* regression coefficient; *rr* respiratory rate; *sd* standard deviation; *ses* sample entropy; *spo2* oxygen saturation

**Fig. 5:**
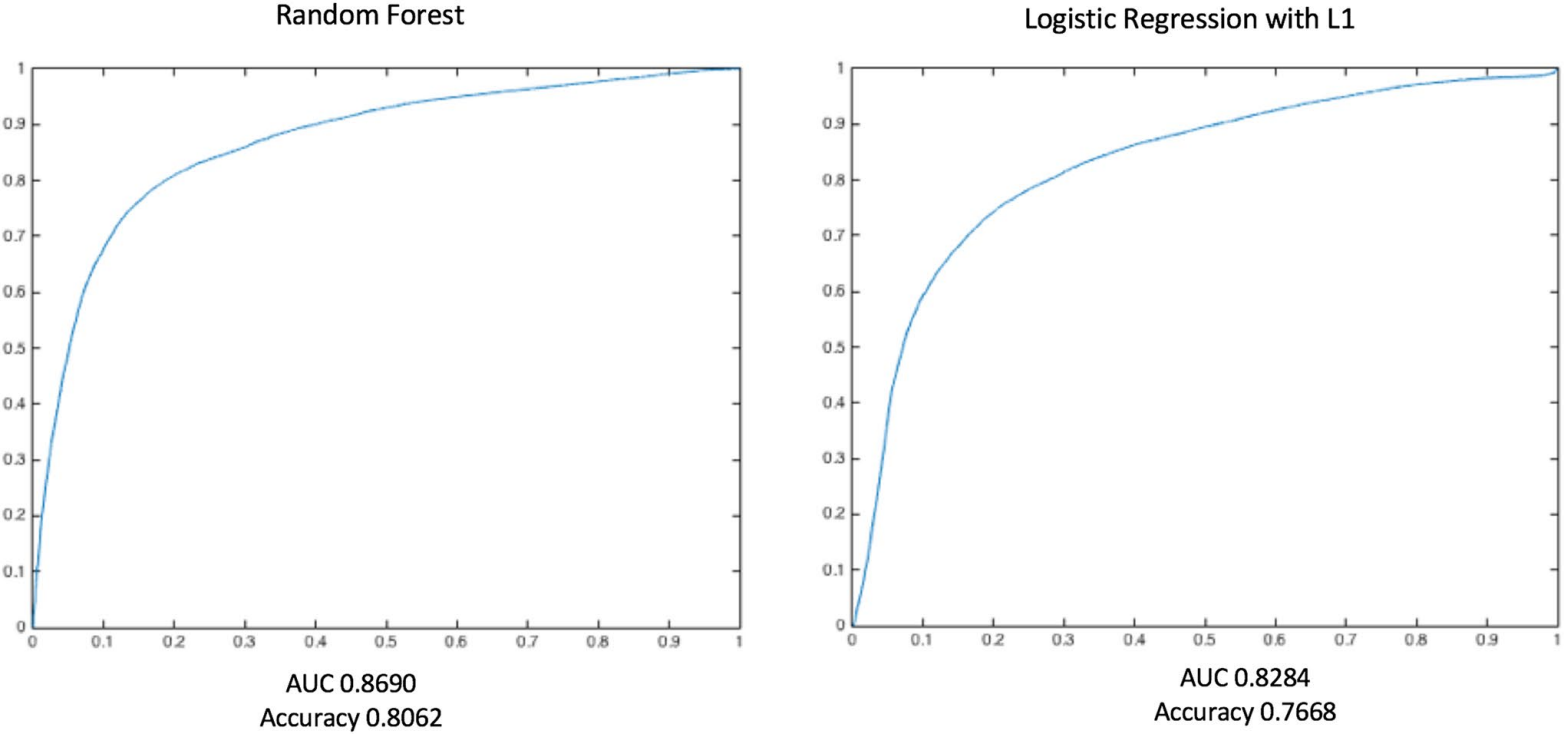
Comparison of the performance of the algorithm. Random Forest (RF, left plot) and Logistic Regression (LR, right plot) with L1 regularization term were tested with using 10-fold cross-validation method. RF slightly outperformed LR with L1 regularization, with overall higher accuracy and larger area under the curve (AUC)

RF performed slightly better than LR regression at lag-0 windows (at the time of tachycardia episode). Cross-validation accuracy of RF ranged from 0.847 (lag 0 min) to 0.782 (lag 30 min), with area under the ROC curve (AUC) ranging from 0.921 to 0.842 (Fig. 5). The average risk scores within the preceding 30 min for control episodes were < 0.3 (< 30% risk of future tachycardia), while the average risk score for case group increased from 0.6 to 0.78 immediately prior to the tachycardia episode. Feature utilization was measured with different prediction horizons of 0, 10, 20, and 30 min before the tachycardia episodes. The top 15 features as shown in Table 3 revealed a mixed set of vital sign representations. While a heavy presence of heart rate-related features was observed to predict future tachycardia, other vital sign features related to respiratory rate and arterial oxygen saturation emerged as the horizon was pushed further ahead of the target episodes.

**Table 3:** Top 15 features for each time period used for different prediction horizons (minutes)

Size of prediction horizon (minutes)	0	10	20	30
Features	aes_hr fft_hr last_10min_mean_hr last_10min_reg_hr last_5min_mean_hr	fft_hr fft_rr last_10min_mean_hr last_5min_mean_rr mean_hr	aes_hr fft_hr fft_rr last_10min_mean_hr last_10min_reg_hr	aes_hr fft_hr last_10min_mean_hr last_10min_mean_rr last_5min_mean_hr
	last_5min_reg_hr mean_hr mean_rr sd_hr ses_hr	mean_rr sd_hr ses_hr aes_spo2 last_5min_mean_hr	last_5min_mean_hr last_5min_mean_rr mean_hr sd_hr ses_hr	last_5min_mean_rr mean_hr sd_hr ses_hr ses_spo2
	reg_hr last_10min_mean_rr fft_rr last_10min_reg_spo2 last_5min_mean_rr	aes_hr mean_abpmean sd_spo2 last_10min_mean_rr reg_hr	mean_abpmean reg_hr mean_rr last_5min_reg_hr last_10min_mean_rr	mean_rr fft_rr reg_rr last_10min_reg_hr last_5min_reg_hr

*Abpmean* mean arterial pressure; *aes* approximate entropy; *fft* fast Fourier transformation; *hr* heart rate; *min* minutes; *reg* regression coefficient; *rr* respiratory rate; *sd* standard deviation; *ses* sample entropy; *spo2* oxygen saturation

### Risk score trajectories

**Fig. 6:**
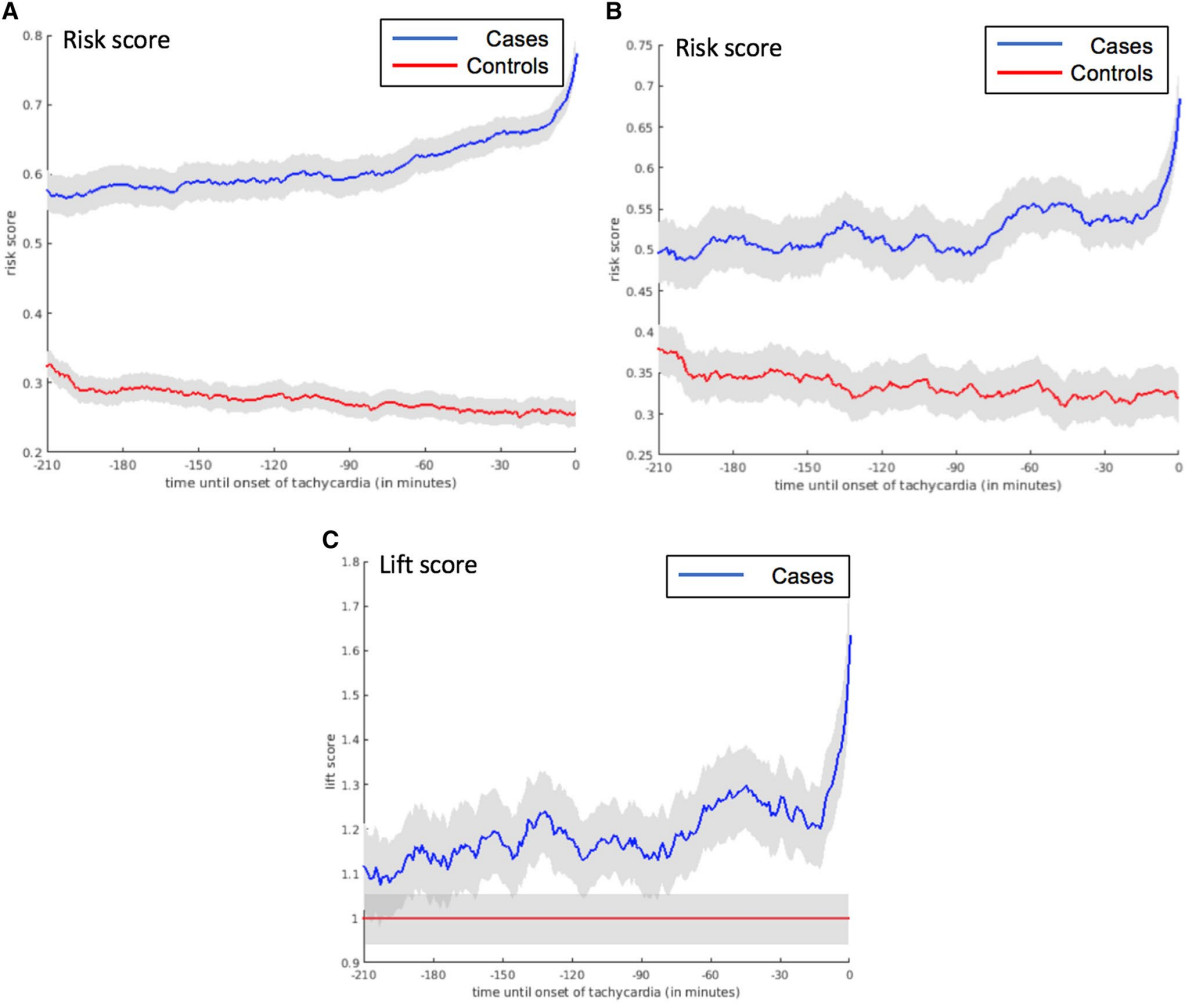
Risk score trajectories for the three models of tachycardia group and control group comparison. **a** Evolving risks for any tachycardia episode in the future. Number of cases = 787, number of controls = 707. **b** Evolving risks for the first tachycardia episode in the future. Number of cases = 240, number of controls = 240. **c** Life score to detect the risk within the same subject prior to the first episode. Number of cases = 235

In comparing the average values of risk for each minute within the evolving trajectory, we first looked at every single episode of tachycardia (n = 787) matched with the corresponding period in controls (Group 1; n = 707). The number of case and control periods were different because the selection of all tachycardia episodes (n = 787) from the case subjects (n = 240) were performed, with the same methodology applied to extract all possible stable non-tachycardia periods (n = 707) from the same number of control subjects. The minute-by minute group averages of the risk score trajectories for the tachycardia and control groups revealed a clear difference in evolution from the beginning of the observation time (3 h prior to the episode), which continued to diverge with time moving forward. When only the first episodes of tachycardia (n = 240) were matched with control periods (Group 2; n = 240), the graph exhibited wider confidence intervals because of smaller sample size. Trajectories were closer to each other compared to the first group especially at the initial period of observation time, and moved further away from the onset of the first episode of tachycardia. Lastly, when the control periods were selected from the same case subjects, the evolving score trajectories (Group 3; n = 235) showed a majority of area overlapping between the case and internal control periods. The difference in size between Group 2 (n = 240) and Group 3 (n = 235) occurred because not all the subjects had at least 3 h of stable baseline periods prior to the pre-tachycardia periods to be able to calculate a baseline risk. The discrimination of the baseline period and pre-tachycardia periods were not possible initially, but the divergence occurred at approximately 75 min prior to onset of tachycardia (the first episode of tachycardia) (Fig. 6).

## Discussion

Using featurized high-density vital sign data, we developed a risk score for predicting tachycardia episodes, which was strongly associated with clinically relevant adverse downstream outcomes. The risk score was generated by a RF algorithm using 30-min rolling windows of featurized data. We used three different modeling groups approaches, and found that: (1) as a whole, risks scores of subjects developing tachycardia were consistently higher than subjects who never developed tachycardia, (2) the first episode of tachycardia is easiest to predict and, (3) patient’s relative risk of developing a tachycardia episode increase as early as 75 min prior to the episode, irrespective of baseline risk.

Episodic tachycardia is multifactorial in its origin and many such episodes may not be related to CRI, although significant and/or persistent tachycardia could herald upcoming organ insufficiency [1, 16, 23], which requires immediate medical response [31]. Timely recognition of these early signals of CRI is critical, as even a short period of organ hypoperfusion during controlled clinical setting can lead to serious adverse outcome including mortality [32]. In line with those prior findings, we found tachycardia subjects who met our operational definition had increased rate of adverse intra-ICU outcomes including vasopressor use, longer ICU stay and increased in-hospital mortality. In addition, the actual number of episodes acutely treated for abrupt non-sinus tachycardia was relatively small. This could imply many non-urgently treated tachycardia episodes could still be related to important prognostic factors, which the algorithm was able to predict. This supports our decision to group all tachycardia as a single modeling target, given our intention to predict overall adverse disease progression, and not specific mechanisms of tachycardia.

Our feature set was designed to incorporate various advanced physiologic time series characteristics including sample and approximation entropy [33], autocorrelation [34], fast Fourier transform [35], density of records, and other regression coefficients, along with traditional statistical representations of numerical vital signs. These multivariate vital sign features were created to infer intricate physiologic crosstalk among cardiovascular and respiratory systems, such potential clinical scenarios include respiratory failure where enhanced cardiovascular activity compensating for hypoxia, or sepsis where respiratory drive increases for the metabolic derangement. Our results showed the utility of features related to respiratory rate and oxygen saturation were increased when the prediction horizon was expanded (Table 3), indicating a combined use of different vital sign features could be an important strategy for earlier prediction of tachycardia. This is of special interest, as the features other than the heart rate itself were strongly predictive of upcoming tachycardic episodes.

The performance of RF was slightly superior to LR with L1 regularization, and this is in concordance with our previous and other studies with retrospective and prospective databases [36–38]. RF is different from other ensemble models due to the introduction of randomness to both observation and its features, robust in datasets with unbalance or missing values and collinearity [25]. This strength fitted well with MIMIC2 data where signals were often missing due to bedside mechanical issues (disconnect from monitor, sensor malfunction), daily patient care (transfer for diagnostic testing, in-bed movement, out-of-bed physical therapy), and loss during pre-processing of the acquired data [39].

Building a risk score trajectory has a practical advantage in quantifying the time-dependent dynamic change in predicting tachycardia from seemingly stable subjects. In addition to predicting all tachycardia episodes (Group 1), we sought to demonstrate whether more clinically relevant scenario could be introduced. Thus, prediction of the first tachycardia episode (Group 2) was attempted, as the subsequent signals of tachycardia could share common etiologies with the first episode and only intensify the gravity of the upcoming CRI. The third control (Group 3) was designed to reflect real-life scenarios, as the initial observational period of non-tachycardia preceded the beginning of tachycardia episode within the same individual. This approach, however, further decreased the sample size because not every patient had enough observational period ahead of the tachycardia episode to be collected. The resultant lift score trajectory demonstrated divergence of risks at around 75 min prior to the tachycardia onset, as shown in Fig. 6c. Based on this prediction, clinicians could potentially have enough time to prepare for upcoming serious tachycardia with preemptive diagnostic and therapeutic approaches to prevent adverse outcomes on an etiology-specific basis. This type of short-term dynamic risk prediction cannot be performed by using traditional risk score systems such as MEWS or NEWS. Still, one should exercise caution not to interpret our predictive graphs only by its large separation from control groups, because a large variance could potentially exist at an individual level trajectory. Therefore, both the absolute value of the risk score and its evolving risk score need to be considered to correctly quantify the risk. Further risk score clustering analysis might help eluding the phenotypic characteristics of individuals developing tachycardia and eventual CRI.

Our study has several limitations. The dataset was not fully utilized to demonstrate the best possible classification performance. The data granularity we used was 1/60 Hz (once a minute), as the matching high-density waveform data could not be effectively featurized due to technical obstacles such as timestamp mismatching and data sparsity. Using an updated version of MIMIC (MIMIC-III) [40], this could possibly be overcome in future extensions of our work. MIMIC-2 was generated more than 10 years ago, making the data less reflective of current clinical practice. For example, the preferred vasopressor for shock has been changed in 2009, which might have affected the outcome of some of the subjects. An observational time window of 30-min was arbitrarily generated. Employing a longer time window would allow accrual of a proportionally larger amount of data with possibly a prediction model that can predict tachycardia with a longer lead time. A much shorter time window would likely have generated models with higher ROC values, but short lead time. Since we planned to create a parsimonious model that could have practical usage in daily patient care, balancing between obtaining larger amount of data to increase lead time and high discrimination at the cost short lead time was an important factor. The modeling work could also benefit from a more exhaustive list of features, and the use of features engineered using existing physiological knowledge. This is particularly relevant if sampling frequency is increased to 1 Hz or above. For example, the predictive value of entropy-based and spectral features would be expected to increase with higher granularity. We note that fast Fourier transforms are highly correlated with the mean value of the variable being featurized, in view of the fact that the computation of total power did not exclude the first component of the transformed variable.

More direct targets such as hypotension, hypoxia, or individual organ failure markers could also be investigated to create a true unifying ‘instability prediction algorithm’. Although ‘significant decompensation associated with shock’ should eventually be defined with clinical correlation, our findings suggest that predicting tachycardia could increase clinical awareness of a higher risk of future hypotension and subsequently other forms of CRI. Additional work is required to refine the specificity of tachycardia predictions and their implications towards development of CRI. Although we presumed these dynamic measures predict tachycardia better than current early warning metrics, we did not directly compare our model’s performance to conventional scoring systems. One reason for that is, there is no practical predictive model for tachycardia in current practice to compare with. Still, others have shown that such static scoring systems are primarily useful for group data and more generalized longer-term predictions. Lastly, we have not assessed the model performance by using external validation cohort in this study, as one of the objectives of this study was providing a roadmap from model development to internal validation. We plan to utilize MIMIC III or other datasets as a potential source for external validation set in future studies with the full use of waveform data.

## Conclusions

Tachycardia, a surrogate marker for CRI, can be predicted with using supervised machine learning algorithms from an intermittent numeric vital sign dataset, and evolving risk of episode could be projected with risk score trajectories. Future research should include identifying and predicting more direct target for CRI, characterization of risk score trajectories towards CRI, and prospective studies to measure clinical outcome changes after the implementation of a risk score triggered treatment approach.
